# Proton spectroscopy of the thalamus in a homogeneous sample of patients with easy-to-control juvenile myoclonic epilepsy

**DOI:** 10.1590/0100-3984.2016.0086

**Published:** 2017

**Authors:** Claudia da Costa Leite, Kette Dualibi Ramos Valente, Lia Arno Fiore, Maria Concepción García Otaduy

**Affiliations:** 1 PhD, Department of Radiology and Oncology, Faculdade de Medicina da Universidade de São Paulo (FMUSP), São Paulo, SP, Brazil.; 2 PhD, Department of Psychiatry, Faculdade de Medicina da Universidade de São Paulo (FMUSP), São Paulo, SP, Brazil.

**Keywords:** Myoclonic epilepsy, juvenile, Magnetic resonance spectroscopy/methods, Thalamus/abnormalities, Epilepsia mioclônica juvenil, Espectroscopia de ressonância magnética/métodos, Tálamo/anormalidades

## Abstract

**Objective:**

Juvenile myoclonic epilepsy (JME) is a subtype of genetically determined generalized epilepsy that does not present abnormalities on conventional magnetic resonance imaging. The aim of this study was to identify metabolic alterations in the thalamus in a clinically homogeneous sample of patients with easy-to-control JME, using short-echo time proton magnetic resonance spectroscopy (MRS).

**Materials and Methods:**

We performed single-voxel (2 cm × 2 cm × 2 cm), short-echo time (TE = 35 ms) proton MRS of the thalamus in 21 patients with JME and in 14 healthy age-matched controls. We quantified N-acetylaspartate (NAA), total NAA, creatine (Cr), choline, and myo-inositol (MI), as well as the sum of glutamate and glutamine signals, all scaled to internal water content, and we calculated metabolite ratios using Cr as a reference. Values of *p* < 0.05 were considered significant.

**Results:**

The MI level and the MI/Cr ratio were significantly lower in the thalami of patients diagnosed with JME than in those of the controls. Other metabolites and their ratios did not differ significantly between the two groups.

**Conclusion:**

In our sample of 21 JME patients, we identified lower levels of MI in the thalamus. No significant abnormalities were observed in the concentrations or ratios of other metabolites.

## Abstract (pt)

**Objetivo:**

A epilepsia mioclônica juvenil (EMJ) é um dos subtipos da epilepsia generalizada geneticamente determinada que não apresenta alterações na ressonância magnética convencional. O objetivo deste estudo foi determinar se há alterações metabólicas no tálamo de pacientes com EMJ de fácil controle de uma amostra clinicamente homogênea utilizando espectroscopia de prótons por ressonância magnética (ERM) com tempo de eco curto.

**Materiais e Métodos:**

Nós realizamos ERM com voxel único (2 cm × 2 cm × 2 cm) e tempo de eco curto (TE = 35 ms) no tálamo de 21 pacientes com EMJ e 14 controles saudáveis pareados por idade. N-acetil-aspartato (NAA), NAA total, creatina (Cr), colina, mio-inositol (MI) e a soma de glutamato e glutamina foram quantificados em relação ao conteúdo de água interna e as razões dos metabólitos foram calculadas utilizando Cr como referência. Valor de *p* < 0,05 foi considerado como significante.

**Resultados:**

Houve redução estatisticamente significante de MI e MI/Cr no tálamo dos pacientes diagnosticados como EMJ em relação aos controles. Outros metabólitos e suas razões não apresentaram alterações significantes.

Conclusão: No tálamo do nosso grupo de 21 pacientes com EMJ foi observada redução de MI e da relação MI/Cr. Não foi observada diferença nos outros metabólitos ou suas relações.

## INTRODUCTION

Juvenile myoclonic epilepsy (JME) is currently classified as genetically determined idiopathic generalized epilepsy (IGE), with a prevalence of 5–11% among all patients with epilepsy and 26% among patients with IGE, making it the most common form of IGE in adults^(1)^. This electroclinical syndrome is characterized by myoclonic seizures in all cases, generalized tonic-clonic (GTC) seizures in approximately 90%, and absence seizures in 15–30%. Interictal electroencephalogram (EEG) shows generalized spike and polyspike-and-wave complexes, which can be triggered by intermittent photic stimulation. The onset occurs during adolescence, between 12 and 18 years of age, and there is a slight female predominance. The classical precipitating factors of JME are sleep deprivation, stress, menstruation, fatigue, and alcohol consumption^(2)^.

The few studies that have focused on the long-term prognosis of JME have produced discrepant results. For some authors, JME is a chronic disease requiring lifelong antiepileptic drug (AED) treatment; for others, patients can achieve remission without continuous AED treatment^(3)^.

In JME, previously defined as a “benign” form of epilepsy, personality disorders and traits related to impulse control and difficulty in accomplishing goals have been reported. Neuropsychological studies suggest that JME patients show impaired performance on multiple subtests that evaluate cognitive functions, especially those associated with frontal lobe function. Certain epilepsy variables, especially higher seizure frequency and longer duration of epilepsy, seem to be associated with the development of personality disorders^(4,5)^. One fact that emerges from these data and variable treatment responses is that JME is a heterogeneous epilepsy syndrome^(6)^.

In JME patients, no structural abnormalities are detectable on routine magnetic resonance imaging (MRI) with visual analysis. However, pathological findings of microdysgenesis in gray and white matter have been described in deceased IGE patients^(7)^. In addition, abnormalities have been detected with other MRI techniques such as proton magnetic resonance spectroscopy (MRS), functional MRI, diffusion tensor imaging, positron emission tomography, and *single-photon emission computed tomography*^(8-14)^.

Structural and functional neuroimaging studies point to fronto-thalamo-cortical dysfunction as the major mechanism in JME^(9,11-13,15)^. Volumetric group analyses of MRI scans of JME patients have shown a decrease in thalamic gray matter volume and an increase in frontal cerebrospinal fluid (CSF) volume^(16)^, supporting that concept.

It should be kept in mind that neuroimaging studies of JME tend to evaluate heterogeneous groups of patients, using different AEDs with distinct doses and outcomes. We believe that it is necessary to study groups of JME patients that are more homogeneous, in order to provide a better delineation of this syndrome. Therefore, the aim of this study was to identify metabolic changes in the thalamus, using short-echo time MRS, in a homogeneous group of patients with classical, easy-to-control JME.

## MATERIALS AND METHODS

### Subjects

This study involved patients with JME and healthy age-matched controls, all of whom were submitted to MRI and MRS of the brain. The diagnosis of JME was made by on the basis of patient medical histories and EEG results. In all patients, EEG studies were performed at the time of diagnosis and over the course of the study, using the international 10–20 system of electrode placement. The diagnostic criteria for JME included a history of myoclonic seizures with or without additional GTC or absence seizures. Although not necessary for the diagnosis, neuroimaging studies were performed in all of the patients included in this study, as well as in the controls. Patients and controls were evaluated by a neurologist and a psychiatrist, in order to identify/exclude any controls with psychiatric disorders, as defined in the Diagnostic and Statistical Manual of Mental Disorders, 4th edition, or a history of neurological disorders.

For patients, the inclusion criteria were as follows: having received a diagnosis of classic JME according to the International League Against Epilepsy criteria^(17)^; having normal neurologic examination results; being adherent to treatment; having been under follow-up treatment for at least 10 years; being between 18 and 35 years of age; and having an IQ within the 80–110 range, as evaluated with a comprehensive battery published elsewhere^(5)^. We excluded patients with the following electroclinical subtypes of JME^(18)^: childhood absence epilepsy persisting and evolving to JME; JME with adolescent-onset absence pyknolepsy; and JME with astatic seizures. Patients with a history of brain trauma, neurosurgery, or epilepsy syndromes other than JME were also excluded, as were those with clinical signs of drug intoxication or any other condition leading to cognitive impairment, those diagnosed with a psychiatric disorder, those with an alcohol or drug abuse, and those having undergone any brain-related surgical intervention. The age at the onset of epilepsy ranged from 8 to 18 years of age (mean, 14.9 ± 2.9 years). The duration of epilepsy ranged from 2 to 36 years (mean, 11.3 ± 8.7 years). In all of the patients, the JME was well controlled with an appropriate AED—sodium valproate (VPA)—in low or moderate doses (≤ 1.0 g/day).

From a group of 35 consecutive patients with JME treated at a tertiary care center, 21 were selected as a homogeneous group for this MRS study. Of those 21 patients, 9 were female. The mean age of the patients selected was 25 ± 8 years (range, 16–45 years). All of the patients were in total remission and therefore had not had any seizures in the period prior to enrollment in the study. In each of the patients, conventional MRI sequences revealed no brain abnormalities.

The control group was composed of 14 healthy volunteers, without neurological and psychiatric disorders, who were matched to the patients for age and gender. Of those 14 subjects, 7 were female. The mean age of the control group subjects was 21 ± 6 years.

The study was approved by the Research Ethics Committee of the Faculdade de Medicina da Universidade de São Paulo, in the city of São Paulo, Brazil. All patients or their legal guardians gave written informed consent.

### MRI and MRS

MRI examinations were performed in two 1.5 T scanners running the same software (Signa Horizon LX, version 9.1; GE Healthcare, Milwaukee, WI, USA). Agreement between the two scanners, in terms of the quantitative MRS results, was confirmed using a calibration phantom with known concentrations of metabolites.

The MRI protocol included acquisition of the following images: axial T1-weighted images with a repetition time/echo time (TR/TE) of 466/19 ms; axial T2-weighted images with a TR/TE of 4500/120 ms and an echo train length of 8; and axial fluid-attenuated inversion recovery images with a TR/TE/inversion time of 11002/148/2200 ms. The slice thickness was 5 mm, and the field of view was 24 cm.

**Figure 1:**
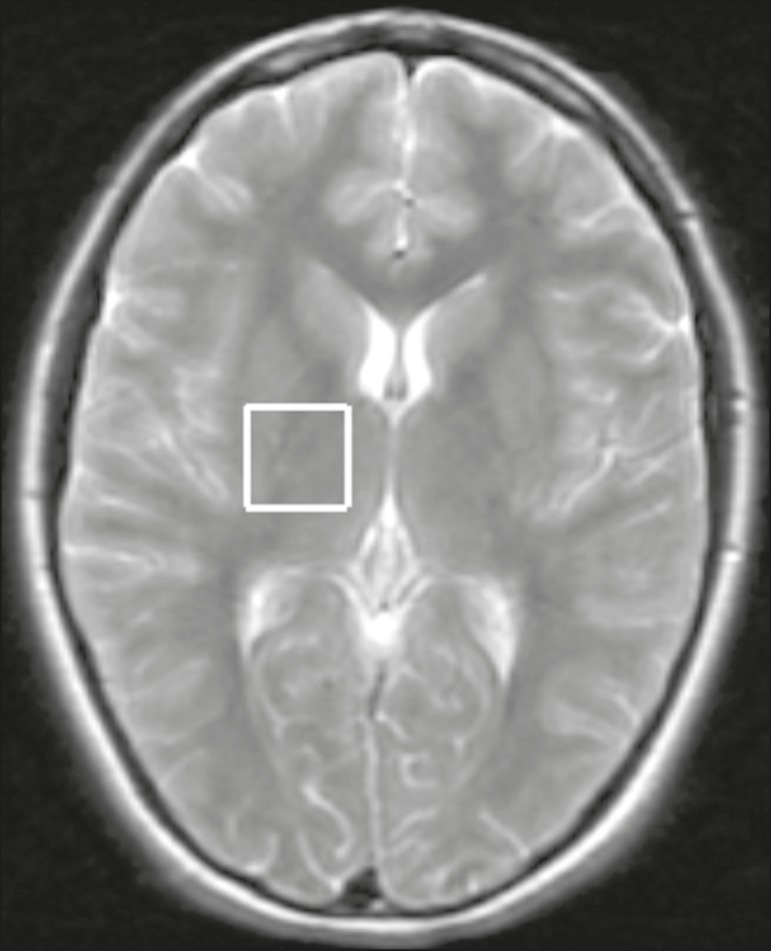
Axial T2-weighted image showing the MRS VOI located on the right thalamus.

Single-voxel MRS of the thalamic region was obtained by positioning a volume of interest (VOI) of 2 cm × 2 cm × 2 cm on an axial slice, as shown in Figure 1. In patients and controls, the MRS VOI was always located on the right thalamus. The MRS technique used was point-resolved spectroscopy (PRESS) with a TR/TE of 1500/35 ms and 8 phase cycling steps. The PRESS sequence was preceded by an automatic pre-acquisition procedure that included adjustment of transmitter/receiver gains, optimization of the flip angle for water suppression, and shimming for the chosen VOI. The water peak line width was never broader than 6 Hz after the pre-acquisition procedure. For each voxel, we acquired 16 unsuppressed water reference acquisitions, followed by 128 water-suppressed acquisitions, while keeping the transmitter and receiver gains constant. Water suppression was achieved with the application of three chemical shift-selective Gaussian pulses. A total of 2048 data points were collected over a spectral width of 2500 Hz. Spectral analysis was performed offline by an experienced spectroscopist, using the LCModel software (Stephen Provencher Inc., Oakville, ON, Canada)^(19)^. Concentration of the following metabolites were evaluated and used for further statistical analysis: N-acetyl-aspartate (NAA) at 2.02 ppm; the sum of NAA and NAA-glutamate (NAAG) at 2.04 ppm, denoted by the symbol tNAA; creatine (Cr)/phosphocreatine at 3.02 ppm; choline-containing compounds (Cho) at 3.22 ppm; myo-inositol (MI) at 3.56 ppm, and the sum of glutamate and glutamine, exhibiting multiple resonances in the regions of 2.1–2.5 ppm and 3.6–3.9 ppm. Signal intensities were scaled to internal water content, and the concentration values obtained (in institutional units) were also used in order to calculate metabolite ratios relative to the Cr resonance. No correction was made for relaxation effects or the contribution of CSF.

### Statistical analysis

Statistical analysis was performed with the IBM SPSS Statistics software package, version 23.0 for Windows 10. The normal distribution of the data was confirmed with the Anderson-Darling test, values of *p* < 0.01 being considered statistically significant. The two-tailed *t*-test was used for comparisons between groups, values of *p* < 0.05 being considered statistically significant. The Levene test for homogeneity of variances was applied.

## RESULTS

**Figure 2:**
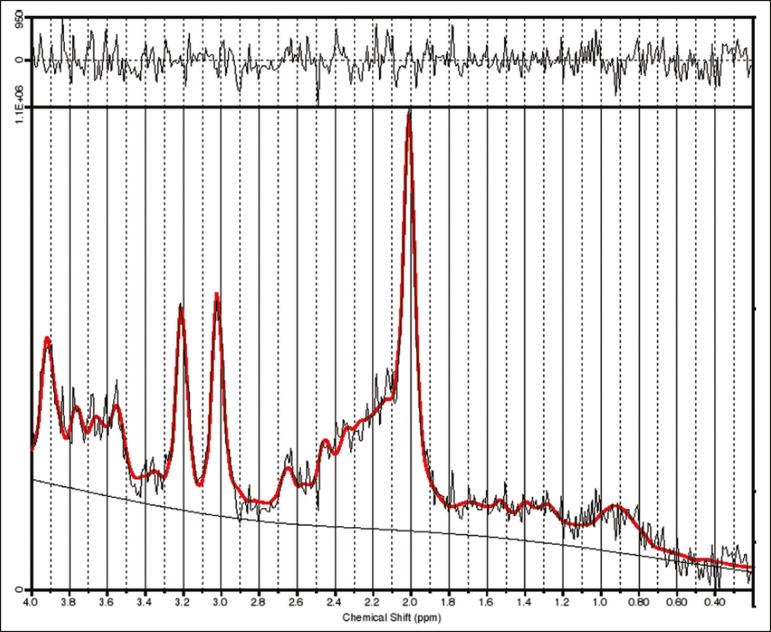
MRS spectrum of the right thalamus, with the fitted spectrum for quantification purposes (red) and the residual spectrum (black).

Figure 2 shows the MRS spectrum of the right thalamus in a patient, with spectral fitting for quantification purposes and the residual spectrum. The mean metabolite concentrations (in institutional units) and metabolite ratios are listed in Tables 1 and 2, respectively. The water linewidths calculated for both groups are shown in Table 2. We found that the MI level and the MI/Cr ratio were lower in the patients than in the control group subjects. No significant differences between patients and controls were observed for any other metabolite concentrations, metabolite ratios, or peak linewidths.

**Table 1:** Mean metabolite concentrations (in institutional units), scaled to internal water content, in JME patients and controls.

	NAA	tNAA	Cr	Cho_t_	MI	Glx
Patients	6.34 ± 0.61	7.73 ± 0.70	4.50 ± 0.34	1.29 ± 0.13	3.00 ± 0.66	9.74 ± 1.10
Controls	6.51 ± 0.79	8.04 ± 0.72	4.45 ± 0.26	1.29 ± 0.14	3.58 ± 0.64	9.74 ± 1.00
*P*	n.s.	n.s.	n.s.	n.s.	0.015	n.s.

NAA, N-acetylaspartate; tNAA, total N-acetylaspartate; Cr, creatine; Cho_t_, total choline; MI, myo-inositol; Glx, glutamate-glutamine; n.s., not significant.

**Table 2:** Mean metabolite ratios, calculated by using Cr as a reference, and mean water linewidths (in Hz), in JME patients and controls.

	LWwater	NAA/Cr	tNAA/Cr	Cho_t_/Cr	MI/Cr	Glx/Cr
Patients	4.24 ± 0.70	1.42 ± 0.18	1.72 ± 0.18	0.29 ± 0.03	0.67 ± 0.15	2.17 ± 0.22
Controls	4.35 ± 1.09	1.46 ± 0.15	1.81 ± 0.17	0.29 ± 0.03	0.81 ± 0.15	2.19 ± 0.25
*P*	n.s.	n.s.	n.s.	n.s.	0.009	n.s.

LWwater, water linewidth; NAA/CR, N-acetylaspartate/creatine ratio; tNAA/Cr, total N-acetylaspartate/creatine ratio; Cho_t_/Cr, total choline/creatine ratio; MI/Cr, myo-inositol/creatine ratio; Glx/Cr, glutamate-glutamine/creatine ratio; n.s., not significant.

## DISCUSSION

Although most MRS studies of JME have demonstrated reduced NAA concentrations or NAA/Cr ratios in the thalamus, the methods applied have varied between single- and multi-voxel measurements, as well as between short and long echo times^(9,10,15,20-24)^. We also found NAA values to be lower in JME patients than in controls, although the difference did not reach statistical significance.

When using short echo times, most authors have shown significantly lower NAA concentrations in the frontal lobe or thalamus of patients with JME^(8,9,15,17,20,21,24)^. Araújo Filho et al.^(10)^ found a lower NAA/Cr ratio in the thalamus. However, when the authors grouped their patients into those with personality disorders (*n* = 16) and those without (*n* = 41), they observed that the patients with personality disorders showed significant lower concentrations of NAA/Cr than did those without. In the present study, we excluded patients with psychiatric disorders, a selection criteria that might explain our findings of normal NAA concentrations and NAA/Cr ratios.

Many MRS studies involving patients with IGE have used clinically heterogeneous groups including the clinical spectrum of childhood absence epilepsy, juvenile absence epilepsy, JME, and GTC seizures. It is possible that the clinical characteristics of the sample influence the results. Our group consisted of patients with JME that was well controlled with low or moderate doses of VPA.

Studies have shown that MI levels are significantly decreased in JME patients^(8,15)^. The only metabolite that was significantly reduced in our group of patients was MI. Savic et al.^(8)^ also found reduced Cho in patients with JME or GTC epilepsy. Although we found no Cho reduction in our sample, we observed a positive correlation between Cho and MI (in the JME patients only) indicating that the MI decrease seems to be accompanied by a Cho decrease in JME. The acquisition technique used in our study is quite similar to that used by Savic et al.^(8)^, the main difference being the voxel size, which was larger in our study, potentially resulting in a larger partial volume effect.

The MI levels can be affected by medications. Two proton MRS studies showed that VPA use in patients with epilepsy causes a decrease in MI levels, not correlated with VPA dose or seizure control, suggesting that the MI decrease is not related to the antiepileptic properties of VPA^(15,25)^. The decrease in cellular MI after sodium VPA administration was previously described in rats by O’Donnell et al.^(26)^. Shaltiel et al.^(27)^ also reported that acute VPA administration decreased MI levels, although the same was not observed for chronic VPA administration. Those authors also showed decreased MI-1-phosphatase synthase in crude human brain cortex homogenate. That enzyme catalyzes the MI formation^(28)^. Hattingen et al.^(15)^ evaluated patients treated or not treated with VPA and observed that the patients using VPA presented more pronounced decreases in MI and NAA in the thalamus. All of the patients in our sample were taking VPA, which could provide a reasonable explanation for the observed decrease in MI.

Savic et al.^(8)^ reported slightly larger linewidths for JME patients than for controls, and the authors attributed that fact to the larger voxel size used in the patients, rather than to real differences between the two groups. In the present study, we found the opposite to be true: the linewidth was found to be slightly smaller in the patients than in the controls, although the difference did not reach statistical significance. It should be noted that in our study the voxel size was kept constant for both groups.

One limitation of our study was that no correction was made for CSF or tissue volume. Nevertheless, the contribution of CSF is not as important in the region of the thalamus as in other regions, such as the cortex. As proof of that, the results did not change when we used Cr, rather than water, as a reference. Another limitation was that the thalamic voxel could include the partial volume of the lentiform nucleus.

The strength of the current study is that all of the patients presented classical, easy-to-control JME. All underwent psychiatric and neuropsychological evaluation; none presented psychiatric disorders or an IQ lower than 80. One may pose the question that our sample does not represent all patients with JME. However, we believe that studies with homogeneous samples are indeed necessary in order to avoid confounding factors. Savic et al.^(8)^ demonstrated that the concentration of NAA was directly correlated with the performance on the Trail Making Test, a neuropsychological paradigm for attention. Subsequently, Araújo Filho et al.^(10)^ observed a correlation between the neuroimaging findings and the presence of personality disorder in patients with JME. There is considerable clinical evidence that JME is heterogeneous and that the subgroups must be addressed carefully, because they could present different outcomes^(3)^. In addition, the presence of a psychiatric disorder and a relevant cognitive deficit might render results not related to the condition itself (JME), but rather to the comorbidity.

In the present study, we did not perform absolute quantification by correcting our concentration values with an external reference. However, we estimated metabolite concentrations using internal water referencing, which in this case was an effective approach, given that water content in JME patients does not differ from that seen in healthy individuals^(8)^.

## CONCLUSION

In patients with classical, easy-to-control JME without psychiatric disorders or mental retardation, MI levels in the thalamus are reduced. Nevertheless, we found no evidence of neuronal damage, as would be reflected by a decrease in NAA levels, underscoring the concept that JME is a heterogeneous disease.

## References

[1] Camfield CS, Striano P, Camfield PR (2013). Epidemiology of juvenile myoclonic epilepsy. Epilepsy Behav.

[2] Wolf P, Yacubian EMT, Avanzini G (2015). Juvenile myoclonic epilepsy: a systemic disorder of the
brain. Epilepsy Res.

[3] Geithner J, Schneider F, Wang Z (2012). Predictors for long-term seizure outcome in juvenile myoclonic
epilepsy: 25-63 years of follow-up. Epilepsia.

[4] Moschetta SP, Valente KD (2012). Juvenile myoclonic epilepsy: the impact of clinical variables and
psychiatric disorders on executive profile assessed with a comprehensive
neuropsychological battery. Epilepsy Behav.

[5] Moschetta S, Fiore LA, Fuentes D (2011). Personality traits in patients with juvenile myoclonic
epilepsy. Epilepsy Behav.

[6] Valente KD, Rzezak P, Moschetta SP (2016). Delineating behavioral and cognitive phenotypes in juvenile
myoclonic epilepsy: are we missing the forest for the trees?. Epilepsy Behav.

[7] Meencke HJ, Janz D (1984). Neuropathological findings in primary generalized epilepsy: a
study of eight cases. Epilepsia.

[8] Savic I, Osterman Y, Helms G (2004). MRS shows syndrome differentiated metabolite changes in
human-generalized epilepsies. Neuroimage.

[9] Lin K, Carrete H Jr, Lin J (2009). Magnetic resonance spectroscopy reveals an epileptic network in
juvenile myoclonic epilepsy. Epilepsia.

[10] Araújo Filho GM, Lin K, Lin J (2009). Are personality traits of juvenile myoclonic epilepsy related to
frontal lobe dysfunctions? A proton MRS study. Epilepsia.

[11] Jiang S, Luo C, Liu Z (2016). Altered local spontaneous brain activity in juvenile myoclonic
epilepsy: a preliminary resting-state fMRI study. Neural Plast.

[12] Keller SS, Ahrens T, Mohammadi S (2011). Microstructural and volumetric abnormalities of the putamen in
juvenile myoclonic epilepsy. Epilepsia.

[13] Kim JH, Suh SI, Park SY (2012). Microstructural white matter abnormality and frontal cognitive
dysfunctions in juvenile myoclonic epilepsy. Epilepsia.

[14] Koepp MJ, Woermann F, Savic I (2013). Juvenile myoclonic epilepsy-neuroimaging findings. Epilepsy Behav.

[15] Hattingen E, Lückerath C, Pellikan S (2014). Frontal and thalamic changes in GABA concentration indicate
dysfunction of thalamofrontal networks in juvenile myoclonic
epilepsy. Epilepsia.

[16] Kim JH, Lee JK, Koh SB (2007). Regional grey matter abnormalities in juvenile myoclonic
epilepsy: a voxel-based morphometry study. Neuroimage.

[17] Berg AT, Millichap JJ (2013). The 2010 revised classification of seizures and
epilepsy. Continuum (Minneap Minn).

[18] Martínez-Juárez IE, Alonso ME, Medina MT (2006). Juvenile myoclonic epilepsy subsyndromes: family studies and
long-term follow-up. Brain.

[19] Provencher SW (1993). Estimation of metabolite concentrations from localized in vivo
proton NMR spectra. Magn Reson Med.

[20] Mory SB, Li LM, Guerreiro CA (2003). Thalamic dysfunction in juvenile myoclonic epilepsy: a proton MRS
study. Epilepsia.

[21] Haki C, Gümüstas OG, Bora I (2007). Proton magnetic resonance spectroscopy study of bilateral
thalamus in juvenile myoclonic epilepsy. Seizure.

[22] Wandscheneider B, Thompson PJ, Vollmar C (2012). Frontal lobe function and structure in juvenile myoclonic
epilepsy: a comprehensive review of neuropsychological and imaging
data. Epilepsia.

[23] Bernasconi A, Bernasconi N, Natsume J (2003). Magnetic resonance spectroscopy and imaging of the thalamus in
idiopathic generalized epilepsy. Brain.

[24] Cevik N, Koksal A, Dogan VB (2016). Evaluation of cognitive functions in juvenile myoclonic epileptic
patients by magnetic resonance spectroscopy and neuropsychiatric cognitive
tests concurrently. Neurol Sci.

[25] Simister RJ, McLean MA, Barker GJ (2007). The effect of sodium valproate on proton MRS visible
neurochemical concentrations. Epilepsy Res.

[26] O'Donnell T, Rotzinger S, Nakashima TT (2000). Chronic lithium and sodium valproate both decrease the
concentration of myo-inositol and increase the concentration of inositol
monophosphates in rat brain. Brain Res.

[27] Shaltiel G, Mark S, Kofman O (2007). Effect of valproate derivatives on human brain
myo-inositol-1-phosphate (MIP) synthase activity and amphetamine-induced
rearing. Pharmacol Rep.

[28] Geiger JH, Jin X (2006). The structure and mechanism of myo-inositol-1-phosphate
synthase. Subcell Biochem.

